# Accuracy and reliability of tibial torsion measurement using radiography and ultrasound in dogs

**DOI:** 10.17221/50/2024-VETMED

**Published:** 2024-12-27

**Authors:** Minseung Jeong, Kyuman Kwack, Jongyeol Kim, Yong Yu, Suyoung Heo

**Affiliations:** Department of Veterinary Surgery, College of Veterinary Medicine, Jeonbuk National University, Jeollabuk-do, Republic of Korea

**Keywords:** intraclass correlation coefficients, non-chondrodystrophic dog, tibial torsion angle

## Abstract

Tibial torsion assessment is crucial for understanding deformities and malalignments that can lead to joint pathologies in dogs. Different methods such as radiography, computed tomography (CT), and three-dimensional (3D) volume-rendering techniques have been employed to measure tibial torsion. This study compared the accuracy and reliability of tibial torsion angle (TTa) measurements obtained using radiography and ultrasound tilting techniques against those obtained using the 3D volume-rendering method in small-to-medium-sized non-chondrodystrophic dogs. Seven dogs with 11 hind limbs were included in this study. Descriptive statistics revealed mean TTa values for radiography (1.6° ± 5.14°), ultrasound (2.92° ± 3.98°), CT (4.57° ± 3.44°), and 3D volume-rendering method (5.29° ± 3.30°). Intraclass correlation coefficient (ICC) analysis indicated excellent intra- and interobserver agreement between the radiography and ultrasound methods. Correlation analysis showed positive correlations between all the methods. These findings demonstrate that radiography and the ultrasound tilting technique are reliable alternatives for measuring TTa. Although slightly lower ICC values were observed than those of the 3D volume-rendering technique, the radiography and ultrasound methods still exhibited good to excellent reliability, suggesting that these alternative methods could be effective diagnostic tools for assessing TTa in clinical settings with high accuracy and reliability.

Tibial torsion, an angular relationship between the transverse axes of the proximal and distal tibial epiphyses about its longitudinal axis, is expressed as external or internal torsion (Paley 2005). Deviations from the normal range, often due to deformity or malalignment, have been reported to cause osteoarthritis and stifle joint pathology in dogs (Fitzpatrick et al. 2012; Dunlap et al. 2016).

A precise method for assessing tibial torsion is essential before corrective osteotomy such as tibial tuberosity transposition in dogs with medial patellar luxation. The realignment of the extensor mechanism in the stifle and repositioning of the patella within the trochlear groove can be corrected by evaluating the degree of tibial torsion and pre-measuring the distance of medial displacement of the tibial tuberosity (L‘Eplattenier and Montavon 2002).

Numerous diagnostic methods have been developed for assessing tibial torsion in humans, including clinical examination, radiography, ultrasound, fluoroscopy, computed tomography (CT), and three-dimensional (3D) computed tomography reconstruction (Clementz 1989; Milner and Soames 1998; Bouchard et al. 2004; Hudson et al. 2006; Guven et al. 2009; Shin et al. 2011; Liodakis et al. 2012; Sangeux et al. 2014; Panou et al. 2015; Borish et al. 2017). Conversely, in veterinary medicine, studies utilise radiography, computed tomography (CT), and three-dimensional (3D) techniques to measure tibial torsion (Apelt et al. 2005; Aper et al. 2005; Petazzoni and Jaeger 2008; Barnes et al. 2015; Savio et al. 2016; Longo et al. 2018; Longo et al. 2019; Longo et al. 2021).

Ultrasound measurements of tibial torsion have proven effective compared to goniometric and CT measurements in humans (Butler-Manuel et al. 1992; Hudson 2008). Additionally, Hudson et al. (2006) demonstrated ultrasound measurement of tibial torsion yields accurate and reproducible results both *in vivo* and *in vitro*.

Slocum and Devine (2000) evaluated tibial torsion in dogs using a radiographic method based on linear measurement (Apelt et al. 2005). Petazzoni and Jaeger (2008) demonstrated a deviation from Slocum’s conventional radiographic method, representing tibial torsion from 5° to 35° as an angle based on the tibial tuberosity’s position. Aper et al. (2005) reported that the radiographic method used by Slocum, employing a linear approach, cannot be directly compared to the CT technique that measures angles. They further stated that the CT technique is more accurate in measuring tibial torsion than the radiographic method due to their lack of susceptibility to minor malpositioning (Apelt et al. 2005). Longo et al. (2021) described that using 3D reconstructed CT images for evaluating tibia torsion provides a more reliable alternative to the CT technique, exhibiting excellent precision and accuracy.

In routine clinical practice, measuring the degree of tibial torsion through CT involves additional costs, risk of anaesthesia, and radiation exposure. Furthermore, ambiguity exists in choosing the axis pair and information regarding breed-specific standards is lacking. The purpose of this study was to 1) compare different commonly used methods of measuring the degree of tibial torsion, 2) examine whether the radiographic assessment method proposed by Petazzoni and Jaeger (2008) and the ultrasound method yield results comparable to those of the 3D volume-rendering method, and 3) determine its clinical applicability.

## MATERIAL AND METHODS

Eleven hind limbs of seven non-chondrodystrophic dogs were examined with owner consent. This study included three dogs affected by unilateral cranial cruciate ligament rupture (CCLR) and four clinically normal dogs. We included eight normal legs and three contralateral normal legs of patients with unilateral CCLR. The limbs were excluded if excessive tibial torsion or rotation was suspected radiographically. The orthopaedic and physical examinations were conducted, and dogs with underlying medical conditions deemed unsuitable for sedation or general anaesthesia, and those afflicted with alternative orthopaedic disorders, were excluded from the study. The limbs included in this study had no surgical history. Skeletally mature dogs were identified by observing the closed physis on radiography.

### Intraclass correlation coefficients (ICC)

The tibial torsion angle (TTa) of each dog was measured by three examiners using four different methods (radiography, ultrasound, CT, and 3D volume-rendering). A professor of small animal orthopaedics (examiner A), an experienced small animal orthopaedic surgeon (examiner B), and a veterinary student (examiner C) participated in the study.

The intraobserver variability was assessed by each examiner, conducting the protocol on three separate occasions to obtain the measurements. The interobserver variability was determined by comparing the measurements taken by examiner A with those taken by examiners B and C.

To maintain evaluation consistency, the sequences of dogs were randomised using a randomisation program (Research Randomizer, v4.0; https://www.randomizer.org/) that varied among examiners. Prior to the study, each examiner conducted practice sessions using unrelated subjects for each evaluation method and was well-acquainted with the protocol.

### Radiographic measurement

**Figure 1:**
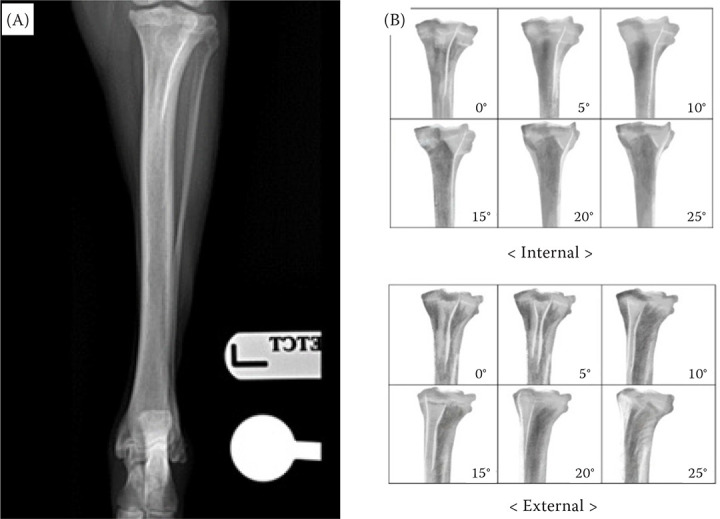
(A) Internal torsion of 5 degrees in the left tibia of a Pomeranian. (B) Degree of tibia tuberosity movement in 5-degree increments Notice the change in the appearance of the tibial tuberosity. Caudo-cranial projection radiograph of the distal tibia showing the medial cortex of the calcaneus transecting the cochlea of the tibia

Craniocaudal radiographs of the stifle, tibia, and tarsus were also obtained. The tibia must be aligned parallel to the X-ray table and longitudinal axis of the tibia while maintaining a craniocaudal position perpendicular to the X-ray beam. The distal tibia was aligned with the medial side of the calcaneus, parallel to the middle of the cochlea in the craniocaudal view. The direction of torsion refers to the assessment of the proximal to the distal aspect of the limb, with the reference point being the position of the distal portion of the limb (Petazzoni and Jaeger 2008). It represents the degree of tibial tuberosity movement in 5-degree increments with a torsion level ranging from 0° to 35°. When internal torsion was present, the tibial tuberosity was positioned on the lateral cortex with 20° as the reference point. In contrast, with external torsion, the tibia tuberosity was placed on the medial cortex, with 20° as the reference point (Figure 1).

### Ultrasound tilting measurement

**Figure 2:**
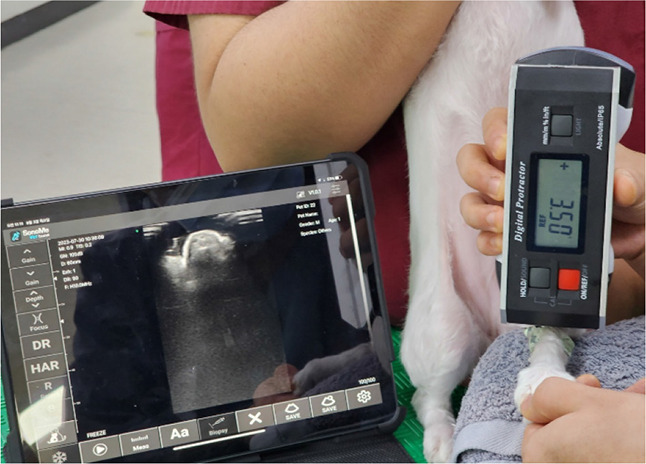
External torsion of 3.5 degrees in the left tibia of a Maltese The white arrow indicates the horizontal view of the cranial margin of the distal tibia

Tibial torsion was measured using a portable unit with a 40-mm linear array transducer (H5C10L; SonoMe, Guro-gu, Seoul, Republic of Korea). The data collection involved utilising frequencies ranging from 7.5 to 10 MHz, with the specific frequency selected based on the depth of the soft tissue between the skin and the bone surface. The inclination angle of the ultrasound transducer required to align the image to the horizontal position of the monitor was measured using a calibrated digital inclinometer (BD-413WP; Bluetec, Guro-gu, Seoul, Republic of Korea). The subjects were placed in a dorsal recumbent position to ensure that the tibia was parallel to the floor surface. A towel was placed on the caudal aspect of the tibia to secure its position and the stifle joint was flexed. The cranial surface of the tibia was positioned upward to align the caudal aspect of the tibial plateau with the floor in a horizontal position. The tilting technique used in a previous study was used in this research (Terjesen and Anda 1987; Miller et al. 1993; Miller et al. 1997; Hudson et al. 2006; Hudson 2008). It is essential to identify the flat surfaces of the proximal and distal tibia using a transducer to measure tibial torsion. The proximal aspect of the tibia was identified using a transducer to locate the caudal tibial plateau. The transducer was tilted until the caudal tibial plateau was aligned horizontally on the monitor and the inclinometer was zeroed to establish the base angle. The distal aspect of the tibia was measured using a transducer, starting from the tarsal joint and moving proximally until the cranial capsule margin became visible. Upon identifying the cranial capsule margin, the transducer was tilted until it was aligned horizontally on the monitor, and the tilting angle was measured (Figure 2).

### Computed tomographic measurement

**Figure 3:**
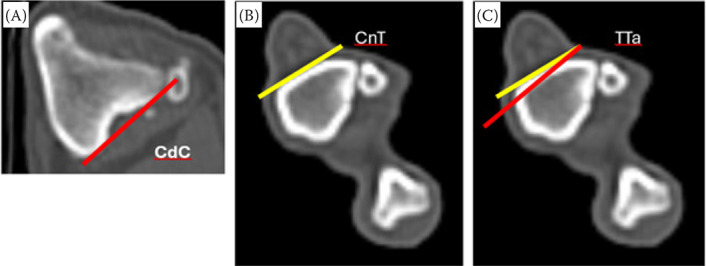
Using an axial computed tomographic (CT) technique for evaluating the tibial torsion angle (TTa) (A) Caudal condylar (CdC) axis means line that parallel to the caudal condylar tibial cortex just distal to the tibial plateau. (B) Cranial tibial (CnT) axis means line running parallel to the cranial tibial cortex and just proximal to the tarso-crural joint. (C) Tibial torsion angle (TTa) defined as the difference between the angle of the caudal condylar (CdC) axis and cranial tibial (CnT) axis

The patients were positioned in dorsal recumbency on a foam cradle, with their hind limbs extended. The tibia was positioned parallel to the CT table. Whole-body CT scans were conducted using a 16-slice helical CT scanner (Toshiba Alexion 16; Toshiba Medical System, Tochigi, Japan) with a slice thickness of 1 mm, in the distal to proximal direction. CT images were assessed utilising a picture archiving and communication system (INFINITT PACS; INFINITT Healthcare Co., Seoul, Republic of Korea) on a diagnostic imaging workstation (ZAL-MAN; Windows7 EnterpriseK, Gyeonggi-do, Republic of Korea). Tibia torsion on CT was measured using the conventional method by Aper et al. (2005). The torsion angle was determined by measuring the angular difference between the axes of the proximal and distal aspects of the tibia. The axes were established based on the anatomical landmarks of the proximal and distal aspects of the tibia. For the CT transverse section of the proximal tibia, the caudal condylar (CdC) axis was described as the line parallel to the caudal condylar tibial cortex, just distal to the tibial plateau. For the transverse CT section of the distal tibia, the distal cranial tibial (CnT) axis was defined as a line running parallel to the cranial tibial cortex and proximal to the tarsocrural joint. The TTa measured by using the “Cobb angle function” was determined by subtracting the angle of the distal cranial tibial (CnT) axis from the caudal condylar (CdC) axis (Figure 3). According to Aper et al. (2005), a positive angle is assigned to the external tibial torsion, whereas a negative angle is assigned to the internal tibial torsion.

### 3D volume-rendering measurement

**Figure 4:**
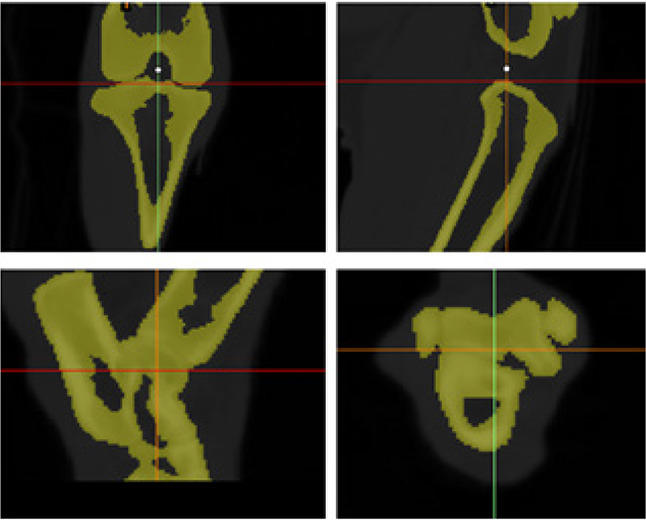
The proximal and distal tibial landmarks required to draw the mechanical tibial axis were found using multiplanar reconstruction (MPR) function

**Figure 5:**
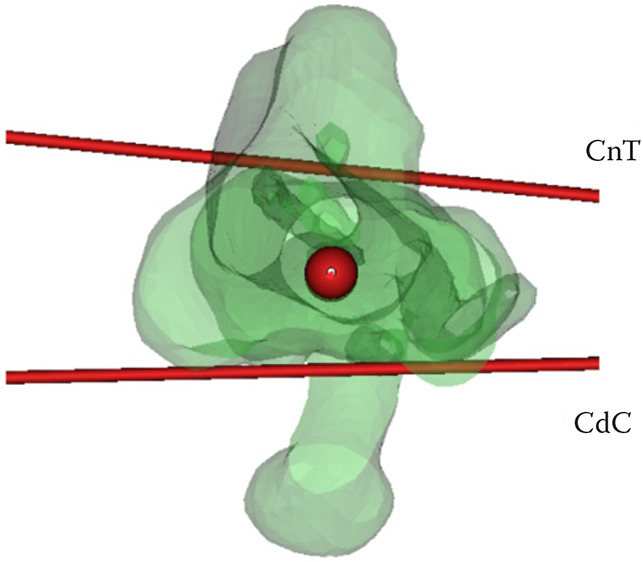
The caudal condylar (CdC) and cranial tibial (CnT) axes were drawn, and the TTa was calculated

The 3D volume-rendering approach proposed by Longo et al. (2021) was applied. The CT’s DICOM images were acquired and converted using Mimics Medical software (Materialise NV, Leuven, Belgium). The mechanical axis of the tibia is represented by two points to establish the central axis. The proximal aspect represents the midpoint between the intercondylar tuberosity and the centre of the proximal articular surface. The distal aspect represents the centre of the distal articular surface and is located at the convex caudal border of the cochlea, serving as the midpoint between the medial and lateral malleoli (Dismukes et al. 2007) (Figure 4). Using the 3D volume-rendering method, the tibia is separated from the surrounding bones including the femur and metatarsus. To establish the caudal condylar (CdC) axis, the most caudal points of the medial and lateral tibial condyles were identified. The distal cranial tibial (CnT) axis was drawn to connect the most prominent points on the distal cranial tibial cortex. To enhance clarity when the proximal and distal planes overlapped, a semitransparent filter was applied to adjust the transparency of the bones, making them easier to visualise. The two points represented by the mechanical axis were aligned to a single point by aligning the axis in the 2D plane. As the caudal condylar (CdC) and distal cranial tibial (CnT) axes exist in the same plane, the Cobb function was used to measure the angle between the two axes and assess the degree of torsion (Figure 5).

### Statistical analysis

The SPSS software package (v29.0.1.0; IBM Corp., New York, NY, USA) was used for data analysis. Data normality was tested using the Kolmogorov-Smirnov test. To evaluate the repeatability, reproducibility, and interobserver reliability of the assessments, an ICC analysis was performed. The ICC grade of Fleiss was used to analyse our results (excellent, > 0.75, good to fair 0.75–0.4, poor < 0.4) (Fleiss 1986). The correlation between the different measurement methods was analysed using Pearson’s correlation coefficient. The results were considered statistically significant at a *P*-value of less than 0.05.

## RESULTS

Eleven tibiae from 11 dogs were used in this study, including five neutered males and two spayed females. The mean age was 5.1 years (median 3.1; range 1.1–10.3). The mean body weight was 5.8 kg (median 4.85; range 2.1–16.7). The breeds included Maltese (2), Chihuahua (1), Pomeranian (3), and mixed breeds (1).

The TTa measured using radiography, ultrasound, CT, and 3D volume-rendering methods are as follows: the mean values of TTa are radiography (1.61° ± 5.14°), ultrasound (2.92° ± 3.98°), CT (4.57° ± 3.44°), 3D volume-rendering (5.29° ± 3.30°). Among these methods, radiography yielded the lowest values, whereas 3D volume-rendering yielded the highest measurements (Table 1).

**Table 1:** Mean and SD values of TT angle by the 3 examiners

TT angle	Examiner A*	Examiner B*	Examiner C*	Examiner A, B, C*
Radiography (°)	0.75 ± 5.46	1.96 ± 5.29	2.12 ± 4.68	1.61 ± 5.14
Ultrasound (°)	2.58 ± 3.68	2.55 ± 4.48	3.63 ± 3.77	2.92 ± 3.98
CT (°)	3.84 ± 3.65	4.95 ± 3.61	4.93 ± 3.02	4.57 ± 3.44
3D CT (°)	4.53 ± 3.63	5.75 ± 3.40	5.59 ± 2.80	5.29 ± 3.30

*Mean ± standard deviation

3D = three-dimensional; CT = computed tomography; TT = tibial torsion

The ICC for intraobserver A, B, and C were as follows: radiography (0.82), ultrasound (0.87), CT (0.92), and 3D volume-rendering (0.96). Although radiography and ultrasound showed slightly lower ICC values than the 3D volume-rendering technique, they demonstrated excellent intraobserver agreement (Table 2). The ICC for interobserver A, B, and C were as follows: radiography (0.90), ultrasound (0.92), CT (0.96); and 3D volume-rendering (0.96). Similar to the intraobserver ICC, the interobserver ICC demonstrated excellent agreement among the examiners (Table 3). All three examiners exhibited excellent ICC in terms of either intraobserver or interobserver agreement.

**Table 2:** Intraobserver ICC measured with radiography, ultrasound, CT, and 3D volume rendering techniques

Technique	Examiner A ICC	Examiner B ICC	Examiner C ICC	Examiner A, B, C ICC
Radiography	0.90 (0.72–0.97)	0.82 (0.53–0.94)	0.75 (0.36–0.92)	0.82 (0.36–0.94)
Ultrasound	0.83 (0.55–0.95)	0.89 (0.71–0.96)	0.89 (0.69–0.96)	0.87 (0.55–0.96)
CT	0.90 (0.74–0.97)	0.94 (0.81–0.98)	0.94 (0.86–0.98)	0.92 (0.81–0.98)
3D CT	0.97 (0.92–0.99)	0.95 (0.81–0.98)	0.98 (0.96–0.99)	0.96 (0.81–0.98)

Data are mean (95% CI)

3D = three dimensoinal; CT = computed tomography; ICC = intraclass correlation coefficient

**Table 3:** Interobserver ICC measured with radiography, ultrasound, CT, and 3D volume rendering techniques

Technique	Examiner A ICC	Examiner B ICC	Examiner C ICC	Examiner A, B, C ICC
Radiography	0.93 (0.84–0.97)	0.91 (0.79–0.97)	0.88 (0.73–0.96)	0.90 (0.73–0.97)
Ultrasound	0.93 (0.84–0.97)	0.90 (0.79–0.97)	0.93 (0.85–0.98)	0.92 (0.79–0.98)
CT	0.96 (0.90–0.98)	0.95 (0.89–0.98)	0.97 (0.93–0.99)	0.96 (0.89–0.99)
3D CT	0.97 (0.93–0.99)	0.96 (0.92–0.99)	0.97 (0.94–0.99)	0.96 (0.92–0.99)

Data are mean (95% CI)

3D = three dimensoinal; CT = computed tomography; ICC= intraclass correlation coefficient

A strong positive linear relationship is observed when the correlation coefficient (*r*) falls within the range of +0.7 to +1.0. In our findings, the correlation between CT and 3D volume-rendering was *r* = 0.953 (*P* < 0.001), demonstrating the strongest positive correlation. Conversely, the correlation between CT and ultrasound was *r* = 0.884 (*P* < 0.001), showing the weakest positive correlation. The correlation coefficient between 3D volume-rendering and radiography was *r* = 0.900, *P* < 0.001, and that between 3D imaging and ultrasound was *r* = 0.893, *P* < 0.001, indicating a positive correlation (Table 4).

**Table 4:** Pearson correlation coefficients of tibial torsion angle measurements by radiography, ultrasound, CT, and 3D-volume rendering techniques

Technique	Radiography	Ultrasound	CT	3D CT
Radiography	1	–	–	–
Ultrasound	0.926***	1	–	–
CT	0.906***	0.884***	1	–
3D CT	0.900***	0.893***	0.953***	1

*P < 0.05, **P < 0.01, ***P < 0.001

3D = three dimensional; CT = computed tomography

## DISCUSSION

In this *in vivo* study, the radiographic method used by Petazzoni and Jaeger (2008) and the ultrasound tilting technique were compared to 3D volume-rendering results to assess the TTa measurement in small-to-medium-sized nonchondrodystrophic dogs.

A previous study evaluated CT and 3D volume-rendering techniques for accuracy by assuming anatomical measurements as the gold standard (Longo et al. 2021). The study demonstrated how closely CT and 3D volume-rendering methods aligned with the gold standard, indicating their precision. The results also indicate that the 3D volume-rendering technique exhibits excellent precision and accuracy. Additionally, CT has been described as a reliable technique with good-to-excellent precision and accuracy. Accordingly, in this study, the 3D volume-rendering technique was adopted as the gold standard, and the accuracy and reliability of the TTa obtained through radiography and ultrasound methods were evaluated and compared with the TTa derived from the 3D volume-rendering method.

CT and 3D volume-rendering methods have certain limitations such as additional costs, time requirements, risks associated with anaesthesia, and radiation exposure. In contrast, radiography and ultrasound tilting techniques offer relative safety and the advantage of not requiring anaesthesia. Based on these research findings, radiography and ultrasound tilting techniques demonstrated a positive correlation with 3D volume-rendering techniques. Additionally, statistical analysis using the ICC showed significant results. Our findings suggest that these techniques have significant clinical applications.

Aper et al. (2005) established the concepts of the CdC/CnT and TC/CnT axes when describing TTa measurement using CT. In previous studies, the CdC/CnT axis was used for medium-to-large-breed dogs, and the TC/CnT axis was used for small-to-medium-sized dogs (Fitzpatrick et al. 2012; Yasukawa et al. 2016; Lusetti et al. 2017; Newman and Voss 2017; Longo et al. 2021). In this study, although small- and medium-sized dogs were included, the CdC-CnT axis was applied for comparison with the ultrasound tilting method.

A negative value was assigned for internal TTa and a positive value for external TTa, consistent with previous research (Yasukawa et al. 2016; Lusetti et al. 2017; Phetkaew et al. 2018). In studies utilising CdC/CnT, the mean values of the angles in medium to large-breed dogs generally showed negative values, indicating internal tibial torsion (Newman and Voss 2017; Longo et al. 2021). On the other hand, studies conducted on small-to-medium breed dogs using TC/CnT typically exhibited mean values of positive (+) values, indicating external tibial torsion (Fitzpatrick et al. 2012; Yasukawa et al. 2016; Lusetti et al. 2017; Phetkaew et al. 2018). In this study, TTa of small-to-medium breed dogs was measured using the CdC/CnT axis, and the mean value was 5.29° ± 3.30°. It can be concluded that among the nonchondrodystrophic normal small-breed dogs, there is evidence of mild external tibia torsion.

The tibial torsion measurement method used in a previous study based on Slocum’s radiographic method employed a linear approach and was not directly comparable to the angle measurement based on the axes in CT images. It was observed that normal canine stifle joints exhibit 15° of internal rotation when extended. Based on this finding, radiographic imaging was performed, with an additional 15° of internal rotation applied. The degree of torsion was defined as the distance between the medial aspect of the calcaneus at the level of the talocrural joint and the base of the talar sulcus. Slocum and Devine (2000) reported that the calcaneus’s medial aspect rotates outward in internal tibial torsion and inward in external tibial torsion (Apelt et al. 2005). In contrast, the radiographic technique used in this study, which was based on Petazzoni’s method, involved positioning the medial aspect of the calcaneus at the middle cochlea of the tibia and observing the altered position of the tibial tuberosity on the radiographic images. Furthermore, this method provides indications within a range of 0° to 35°. It establishes the criteria with radiographic images, such as the tibial tuberosity aligning with the lateral cortex as a reference at 20° for internal torsion and aligning with the medial cortex at 20° for external torsion. However, one limitation of this method is that the angle intervals are spaced 5° apart, making it difficult to determine the TTa within those intervals precisely. This introduced subjective factors into the measurement process. Moreover, considering the anatomical and structural variations across different breeds, it is challenging to universally apply radiographic techniques based on Petazzoni’s method to all breeds (Petazzoni and Jaeger 2008).

To the best of our knowledge, this is the first study in veterinary medicine in which the ultrasound tilting method was employed to incorporate data to compare TTa techniques. When applying the ultrasound tilting method, the caudal tibial plateau can be considered as the CdC axis of the CT measurement. In contrast, the cranial capsule margin of the tibia’s distal aspect can be considered the CnT axis. The reliability of ultrasound for measuring tibial torsion (ICC = 0.93) has been reported in human medicine (Hudson et al. 2006). When using the ultrasound method for TTa measurement, the mean angle was 2.92° ± 3.98° with an intraobserver ICC of 0.87 and an interobserver ICC of 0.92. In the case of TTa, the ultrasound method showed higher values compared to radiography (1.61° ± 5.14°), but lower values compared to CT (4.57° ± 3.44°) and 3D volume-rendering method (5.29° ± 3.30°). The intraobserver ICC was higher in the ultrasound method than in radiography (0.82) but lower than that in the CT (0.92) and 3D volume-rendering methods (0.96). The interobserver ICC was larger than the value for radiography (0.90) but smaller than that for CT (0.96) and the 3D volume-rendering method (0.96). Furthermore, correlation analysis was conducted to examine the relationship with other methods, which revealed a positive correlation. This analysis demonstrated that the ultrasound method is accurate and reproducible for measuring TTa.

High accuracy and reliability provide objectivity to the measurement methods. This study aimed to assess the clinical significance of radiographic and ultrasound methods based on their measured reliability and accuracy. The 3D volume-rendering and CT methods demonstrated similarly high levels of intraobserver and interobserver reliability, consistent with previous research (Longo et al. 2021). The radiography and ultrasound method showed slightly lower interobserver and intraobserver reliability compared to the 3D volume-rendering technique and CT method. However, the values were in excellent agreement with a reliability of 0.75 or higher. These findings suggest that radiography and ultrasound methods can serve as valuable diagnostic tools.

Both the 3D volume-rendering technique and CT-based methods are effective in measuring TTa. However, in terms of accessibility to practical anatomical structures, using cadaveric specimens has been demonstrated to be the most effective approach for anatomical measurements in previous studies (Longo et al. 2021). This is because it facilitates easier axis alignment, simplifying the TTa measurement process. One limitation of this study is the absence of anatomical measurements in the control group.

This study was limited by insufficient sample size, as well as the inclusion of a non-chondrodystrophic heterogeneous group of small-to-medium-sized dogs, which precluded a detailed investigation of tibial torsion angle within a specific breed. Furthermore, since abnormal tibial torsion frequently occurs in patients with stifle joint disorders, such as medial patellar luxation it is important to establish reference ranges through additional research.

In conclusion, the TTa evaluations using the radiographic method of Petazzoni and the ultrasound tilting method are reliable alternatives to the 3D volume-rendering technique and exhibit excellent precision and accuracy. These results suggest that these alternative methods could be effective diagnostic tools for assessing TTa in clinical settings.

## References

[R1] Apelt D, Kowaleski MP, Dyce J. Comparison of computed tomographic and standard radiographic determination of tibial torsion in the dog. Vet Surg. 2005 Sep-Oct; 34(5):457-62. 16266337 10.1111/j.1551-2916.2005.00069.x

[R2] Aper R, Kowaleski MP, Apelt D, Drost WT, Dyce J. Computed tomographic determination of tibial torsion in the dog. Vet Radiol Ultrasound. 2005 May-Jun;46(3):187-91. 16050274 10.1111/j.1740-8261.2005.00048.x

[R3] Barnes DM, Anderson AA, Frost C, Barnes J. Repeatability and reproducibility of measurements of femoral and tibial alignment using computed tomography multiplanar reconstructions. Vet Surg. 2015 Jan;44(1):85-93. 25110206 10.1111/j.1532-950X.2014.12265.x

[R4] Borish CN, Mueske NM, Wren TAL. A comparison of three methods of measuring tibial torsion in children with myelomeningocele and normally developing children. Clin Anat. 2017 Nov;30(8):1043-8. 28470694 10.1002/ca.22894 PMC5647201

[R5] Bouchard R, Meeder PJ, Krug F, Libicher M. Bestimmung der Tibiatorsion – Vergleich von klinischen Winkelmessungen zur Computertomographie [Evaluation of tibial torsion – Comparison of clinical methods and computed tomography]. Rofo. 2004 Sep;176(9):1278-84. German. 15346263 10.1055/s-2004-813366

[R6] Butler-Manuel PA, Guy RL, Heatley FW. Measurement of tibial torsion: A new technique applicable to ultrasound and computed tomography. Br J Radiol. 1992 Feb; 65(770):119-22. 1540801 10.1259/0007-1285-65-770-119

[R7] Clementz BG. Assessment of tibial torsion and rotational deformity with a new fluoroscopic technique. Clin Orthop Relat Res. 1989 Aug;(245):199-209. 2752622

[R8] Clementz BG. Fluoroscopy of rotation in tibial fractures. Acta Orthop Scand. 1989 Apr;60(2):204-7. 2728884 10.3109/17453678909149255

[R9] Dismukes DI, Tomlinson JL, Fox DB, Cook JL, Song KJ. Radiographic measurement of the proximal and distal mechanical joint angles in the canine tibia. Vet Surg. 2007 Nov-Dec;36(8):699-704. 17894597 10.1111/j.1532-950X.2007.00323.x

[R10] Dunlap AE, Kim SE, Lewis DD, Christopher SA, Pozzi A. Outcomes and complications following surgical correction of grade IV medial patellar luxation in dogs: 24 cases (2008–2014). J Am Vet Med Assoc. 2016 Jul 15;249(2): 208-13. 27379597 10.2460/javma.249.2.208

[R11] Fitzpatrick CL, Krotscheck U, Thompson MS, Todhunter RJ, Zhang Z. Evaluation of tibial torsion in Yorkshire Terriers with and without medial patellar luxation. Vet Surg. 2012 Dec;41(8):966-72. 23198923 10.1111/j.1532-950X.2012.01041.x

[R12] Fleiss JL. The design and analysis of clinical experiments. New York: John Wiley & Sons; 1986.

[R13] Guven M, Akman B, Unay K, Ozturan EK, Cakici H, Eren A. A new radiographic measurement method for evaluation of tibial torsion: A pilot study in adults. Clin Orthop Relat Res. 2009 Jul;467(7):1807-12. 19052824 10.1007/s11999-008-0655-z PMC2690742

[R14] Hudson D, Royer T, Richards J. Ultrasound measurements of torsions in the tibia and femur. J Bone Joint Surg Am. 2006 Jan;88(1):138-43. 10.2106/JBJS.D.02924 16391259

[R15] Hudson D. A comparison of ultrasound to goniometric and inclinometer measurements of torsion in the tibia and femur. Gait Posture. 2008 Nov;28(4):708-10. 18555685 10.1016/j.gaitpost.2008.04.017

[R16] L’Eplattenier H, Montavon P. Patellar luxation in dogs and cats: Management and prevention. Compend Contin Educ Pract Vet. 2002;24(4):292-8.

[R17] Liodakis E, Doxastaki I, Chu K, Krettek C, Gaulke R, Citak M, Kenawey M. Reliability of the assessment of lower limb torsion using computed tomography: Analysis of five different techniques. Skeletal Radiol. 2012 Mar;41(3):305-11. 21560009 10.1007/s00256-011-1185-4

[R18] Longo F, Nicetto T, Banzato T, Pozzi A, Contiero B, Isola M. Automated computation of femoral angles in dogs from three-dimensional computed tomography reconstructions: Comparison with manual techniques. Vet J. 2018 Jan; 232:6-12. 29428094 10.1016/j.tvjl.2017.11.014

[R19] Longo F, Savio G, Contiero B, Banzato T, Nicetto T, Isola M. Accuracy of an automated three-dimensional technique for the computation of femoral angles in dogs. Vet Rec. 2019 Oct 5;185(14):443. 31292274 10.1136/vr.105326

[R20] Longo F, Nicetto T, Pozzi A, Contiero B, Isola M. A three-dimensional computed tomographic volume rendering methodology to measure the tibial torsion angle in dogs. Vet Surg. 2021 Jan;50(1):61-70. 10.1111/vsu.13531 33103799

[R21] Lusetti F, Bonardi A, Eid C, de Bellesini AB, Martini FM. Pelvic limb alignment measured by computed tomography in purebred English Bulldogs with medial patellar luxation. Vet Comp Orthop Traumatol. 2017 May;30(3): 200-8. 28474728 10.3415/VCOT-16-07-0116

[R22] Miller F, Liang Y, Merlo M, Harcke HT. Measuring anteversion and femoral neck-shaft angle in cerebral palsy. Dev Med Child Neurol. 1997 Feb;39(2):113-8. 9062426 10.1111/j.1469-8749.1997.tb07393.x

[R23] Miller F, Merlo M, Liang Y, Kupcha P, Jamison J, Harcke HT. Femoral version and neck shaft angle. J Pediatr Orthop. 1993 May-Jun;13(3):382-8. 8496377 10.1097/01241398-199305000-00021

[R24] Milner CE, Soames RW. A comparison of four in vivo methods of measuring tibial torsion. J Anat. 1998 Aug;193(Pt 2):139-44. 9758144 10.1046/j.1469-7580.1998.19310139.x PMC1467830

[R25] Newman M, Voss K. Computed tomographic evaluation of femoral and tibial conformation in English Staffordshire Bull Terriers with and without congenital medial patellar luxation. Vet Comp Orthop Traumatol. 2017 May;30(3):191-9. 28331928 10.3415/VCOT-16-12-0162

[R26] Paley D. Growth plate considerations. In: Paley D, Herzenberg JE, editors. Principles of deformity correction. Berlin: Springer-Verlag; 2005. p. 703-10.

[R27] Panou A, Stanitski DF, Stanitski C, Peccati A, Portinaro NM. Intra-observer and inter-observer errors in CT measurement of torsional profiles of lower limbs: A retrospective comparative study. J Orthop Surg Res. 2015 Apr 17; 10:67. 25971620 10.1186/s13018-015-0200-1 PMC4440600

[R28] Petazzoni M, Jaeger GH. Atlas of clinical goniometry and radiographic measurements of the canine pelvic limb. 2^nd^ ed. Duluth (GA): Merial; 2008. p. 60-71.

[R29] Phetkaew T, Kalpravidh M, Penchome R, Wangdee C. A comparison of angular values of the pelvic limb with normal and medial patellar luxation stifles in Chihuahua dogs using radiography and computed tomography. Vet Comp Orthop Traumatol. 2018 Mar;31(2):114-23. 29534279 10.3415/VCOT-17-05-0067

[R30] Sangeux M, Mahy J, Graham HK. Do physical examination and CT-scan measures of femoral neck anteversion and tibial torsion relate to each other? Gait Posture. 2014 Jan; 39(1):12-6. 23787150 10.1016/j.gaitpost.2013.05.020

[R31] Savio G, Baroni T, Concheri G, Banzato T, Montin E, Zotti A. Computation of femoral canine morphometric parameters in three-dimensional geometrical models. Vet Surg. 2016 Nov;45(8):987-95. 27716955 10.1111/vsu.12550

[R32] Shin SY, Yoon CH, Lee ES, Kim YW, Kim YB. The availability of radiological measurement of tibial torsion: Three-dimensional computed tomography reconstruction. Ann Rehabil Med. 2011 Dec;35(6):673-9. 22506190 10.5535/arm.2011.35.5.673 PMC3309262

[R33] Slocum B, Devine T. Tibial plateau leveling osteotomy certification course. Eugene, OR, Slocum-Enterprises, Inc., March 3-5, 2000.

[R34] Terjesen T, Anda S. Femoral anteversion in children measured by ultrasound. Acta Orthop Scand. 1987 Aug;58(4): 403-6. 3314318 10.3109/17453678709146366

[R35] Yasukawa S, Edamura K, Tanegashima K, Seki M, Teshima K, Asano K, Hayashi K. Evaluation of bone deformities of the femur, tibia, and patella in Toy Poodles with medial patellar luxation using computed tomography. Vet Comp Orthop Traumatol. 2016 Jan;29(1):29-35. 26638694 10.3415/VCOT-15-05-0089

